# Adaptation of Non-Invasive Cancer Cells to 3D Collagen I Microenvironment Induces Transcriptional Reprogramming Accompanied by a More Complex RNA Landscape

**DOI:** 10.3390/ijms27156907

**Published:** 2026-08-01

**Authors:** Theresa Wießner-Kroh, Stefanie Hübschmann, Gudrun Marquardt, Jennifer Szczesny, Miriam Faxel, Stefan Rubner, Ioannis Papasotiriou

**Affiliations:** 1 Research Genetic Cancer Centre (RGCC) Central Europe GmbH, Weinbergweg 22, D-06120 Halle (Saale), Germany; 2 Research Genetic Cancer Centre (RGCC) International GmbH, Baarerstrasse 95, CH-6300 Zug, Switzerland

**Keywords:** 3D cell culture, collagen I, microenvironment, transcriptional reprogramming, long-term transcriptomic profiling, transcriptomic complexity, protein-coding RNAs, lncRNAs, CWC15

## Abstract

Nowadays, most cancer research still depends on traditional cell culture in Petri dishes or cell culture flasks which do not have the ability to mimic physiological-like conditions in vitro. However, the behavior of cancer cells strongly relies on the interaction with their extracellular microenvironment. Consequently, current advanced approaches focus on three-dimensional (3D) cell culture to overcome such limitations and to enable a better understanding of fundamental processes including cancer development, progression, apoptosis and invasion. However, transcriptional adaptation to and temporal stability within an in vitro 3D microenvironment still appear to be remarkably understudied. In our study, we compared the cellular behavior and whole transcriptome gene expression of three frequently used non-invasive cancer cell lines (HCT-116, A549 and T47D), embedded within a collagen I (Coll I)-based 3D microenvironment to its counterparts grown as simple monolayers in a time-dependent manner. Thereby, changes in morphology and doubling time became apparent between both cultivation systems, and RNA sequencing-based transcriptome-wide analysis revealed a remarkable increase in transcriptional complexity under 3D conditions. In line with the 3D-dependent phenotype, unidirectional shifts for genes involved in cell cycle regulation (e.g., *CCNB1*, *CCNB2*), cell–matrix interaction (e.g., *ADAM8*, *ITGA2*) and metabolic signaling (e.g., *HK2*, *ENO2*) were identified over time, being either activated or repressed. Interestingly, all three cell lines cultured in Coll I matrices displayed a highly distinct RNA content and composition, along with a significantly increased number of expressed protein-coding genes (increase of 3–6%) as well as long non-coding RNAs (increase of 26–48%), suggesting a more multifaceted transcription profile under 3D conditions. Our work clearly highlights that an in vitro 3D Coll I-based cell culture system has an incisive cell-specific impact on the whole transcriptome on a qualitative and quantitative level. This tremendous transcriptional reprogramming implies essential changes in gene regulatory networks and affects phenotypic cancer cell behavior, which should be considered when focusing on downstream applications.

## 1. Introduction

3D cell culture systems have been improved during the last decade to mimic more physiological-like conditions for cell culture-based approaches within the cancer research field [1,2,3]. However, the vast majority of experimental findings still rely on cells grown as flat-shaped monolayers on stiff plastics, which is hardly comparable to an in vivo-like situation. In living organisms, cells are in close contact and in continuous interaction with neighboring cells and their extracellular matrix (ECM). This highly dynamic network represents the structural framework for cellular microenvironments and provides biochemical and mechanical support to cells, tissues, and entire organs [4,5,6]. Vice versa, the embedded cells have a considerable impact on their microenvironment. For instance, matrix remodeling can be induced by cellular ECM receptor-mediated signaling, resulting in alterations of microenvironmental properties like viscoelasticity, topology, or liberation of ligands [7]. This constant bidirectional interaction with the surrounding matrix regulates multiple cellular functions in a spatial and temporal manner, including gene expression, proliferation, apoptosis, migration, and cell–cell communication [1,3,6].

Cancer cells strongly modulate their surroundings to promote cancer development, progression and metastasis [6,8]. Hence, to study cancer cell function it is highly essential to provide functional in vitro microenvironments. As the most abundant protein and one of the main structural components of the ECM in humans, Coll I appears to be an advantageous biopolymer to generate 3D matrices. It serves as an adhesion ligand for cells and exhibits binding capabilities for signaling molecules. Furthermore, it is highly compatible with a variety of mammalian cells due to its natural origin, which features the absence of toxicity as well as low immunogenicity and antigenicity [6]. Several studies describe the cultivation of primary or patient-derived cells in Coll I, in which cells better reflect their physiological phenotype. For instance, cells exhibit self-organization [9,10], retain their capacity to differentiate [11,12], or display gene and protein expression patterns that reflect the original tissue properties [9,10]. Coll I 3D microenvironments offer multiple application options. Next to the phenotypical characterization of cells, the cell organization and their interaction with the matrix as well as functional assays addressing invasiveness of cells can be performed under 3D conditions, being impossible for standard cell culture.

Previous studies comparing cells grown as monolayers to Coll I-embedded cells revealed significant effects on cell growth [13,14], morphology [13,15,16], and metabolism [17]. However, the transcriptomic landscape of cells cultivated in Coll I still remains understudied. Most reports investigated differential gene expression only for single genes. For instance, RNA and protein expression analyses demonstrated an upregulation of several selected genes involved in stemness, cell cycle regulation, and epithelial–mesenchymal transition (EMT) for collagen cultured relative to traditionally grown glioma cell lines [13]. Insulin-producing cells showed an upregulation for genes related to pancreatic function and endocrine differentiation [18]. A similar effect was described for extracellular matrix- and tendon-associated genes when human adipose stromal cells were grown in Coll I hydrogels [11]. Moreover, immune response-related gene expression was affected in induced dendritic cells upon exposure to Coll I matrices [19]. For HepaRG and Huh-7 cells, transcriptome-wide analyses were described revealing either an upregulation of transcripts associated with liver metabolism [17] or cell–matrix interaction and positive regulation of cell differentiation [20], respectively. All these studies report a significant shift in gene expression patterns associated with specific cellular functions. Alternative 3D approaches based on laminin-rich matrices or a mixture of collagen and Matrigel connected an altered cell growth and metabolism with transcriptome-wide changes for cell lines derived from colon or breast cancer [21,22,23]. Moreover, A549 cells and immortalized primary epithelial cells BEAS-2B embedded in Matrigel demonstrated a 3D-induced spherical morphology accompanied by gene expression alterations at the transcriptome level upon 5 days. Terms connected with cell–matrix interaction were promoted in both cell types, whereas immune response was activated only in A549 compared to 2D [24]. These studies mainly focused on the evaluation of protein-coding transcripts, although messenger RNA only accounts for approx. up to 7% of all transcribed RNAs [25,26]. However, non-coding transcripts such as long non-coding RNAs (lncRNAs) came gradually into the spotlight of cancer research during the last decades, uncovering regulatory functions for several cellular and cancer-related processes [27,28,29,30]. Interestingly, differential regulation of lncRNAs was demonstrated for BT-474 spheroids [31] and for MCF10A cells cultured in Matrigel [32]. These lncRNAs are involved in regulating key processes in cancer cells, including cell proliferation, apoptosis, and metastasis. *MALAT1* is a prominent lncRNA associated with cancer-related processes. It directly regulates the expression of *MDM4* by sponging miR-185-5p in A549 and H460 cells [33]. Only a few studies have examined gene expression patterns of lncRNAs in an advanced cell cultivation system so far. Therefore, further research is highly needed to identify common patterns [27].

Differential gene expression mediated by 3D cultivation can also be translated into adjusted cellular function. For instance, breast cancer cell lines grown in different 3D culture systems displayed altered drug sensitivities [21,34,35]. Moreover, higher inhibitory concentrations of chemotherapeutic drugs for treated A549 spheroids were reported to be more comparable to patient-derived data [36]. This is also in line with breast cancer patients displaying a higher correlation with 3D-cultured than with 2D-cultured breast cancer cell lines on transcriptomic level [21], and comparable methylation patterns as well as miRNA expression were described for spheroids derived from colorectal cancer cell lines and FFPE-fixated cancer patient samples [37]. All these data clearly highlight the importance of 3D-derived data integration as an essential part of cancer medicine research.

Although 3D cancer research is a fast-developing field, a comprehensive transcriptome-wide analysis of embedded cancer cells over a longer time period evaluating induced reprogramming and differential gene expression of coding and non-coding RNAs is currently lacking. Therefore, our study characterizes the non-invasive cancer cell lines HCT-116, A549 and T47D, grown in 2D and 3D Coll I-based matrices, regarding their morphology, growth properties, and transcriptome-wide gene expression profile. All three cell lines were derived from different cancer origins but share the common feature of non-invasiveness. Differences in gene expression between invasive and non-invasive cells have been described and might directly impact overall gene expression [21,38]. By exploring the temporal transcriptomic landscape with regard to different RNA classes and differential gene expression of cellular key processes, our work contributes to the fundamental understanding of the multiplex gene regulation of more complex cellular models.

## 2. Results and Discussion

### 2.1. Coll I-Embedded Cancer Cells Demonstrate a Distinct Cell Morphology and Are Significantly Delayed in Their Proliferation

**Figure 1:**
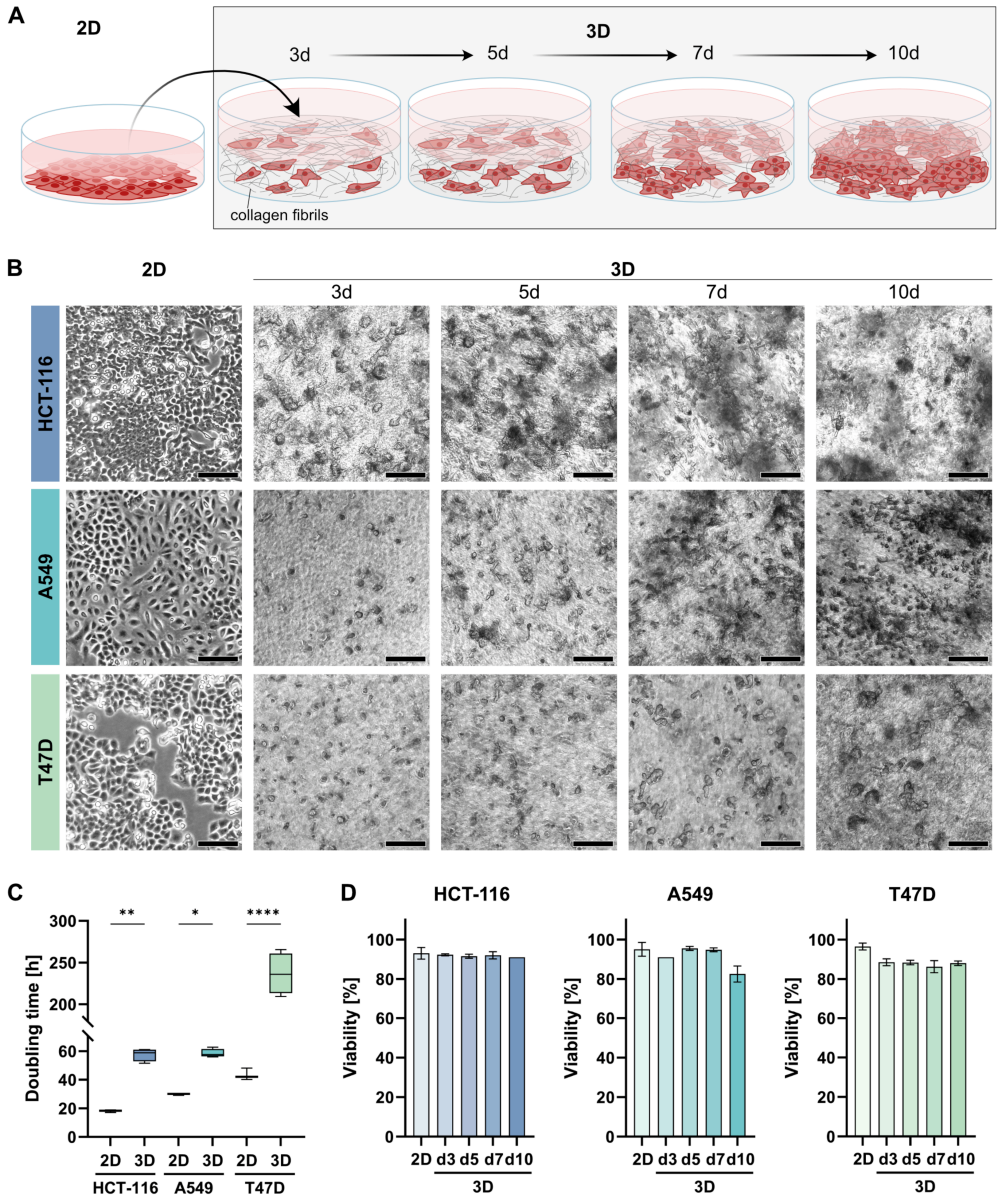
Effects of 3D cultivation on HCT-116, A549 and T47D morphology, proliferation and viability. (**A**) Schematic overview of the experimental setup. 2D-grown cells were embedded into Coll I matrices and further cultivated for 3 to 10 days for subsequent analysis. (**B**) Representative bright-field images of HCT-116, A549 and T47D cells cultivated in Coll I matrices for indicated time periods in comparison to cells grown subconfluently in 2D (scale bar: 200 µm, 4× objective). (**C**) Doubling time determination (*n* = 4). Statistical significance was calculated using Students *t*-test. The *p*-values are indicated by asterisks (* *p* < 0.05, ** *p* < 0.01, **** *p* < 0.0001). (**D**) Determination of cell viability by automated bright-field image analysis (*n* = 4). Data are shown as the mean values ± SD.

To investigate the impact of 3D cultivation conditions on the cellular phenotype of HCT-116, A549 and T47D, cells were embedded in Coll I matrices and cultivated up to 10 days (Figure 1A). The Coll I concentration of 2.5 mg/mL was chosen based on pre-experiments including the analysis of fibrillation kinetics (Figure S1) to ensure that the fibrillation did not start prematurely during the preparation. We further examined the distribution of the cells within the matrix and chose 2.5 mg/mL as the concentration with the most homogenous cell distribution.

In contrast to 2D standard cell culture, distinct morphological changes were observed under 3D conditions, as each cell line lost its flat shape and displayed a more roundish to physically outstretched morphology. When grown on a rigid plastic surface, cells artificially exhibit monolayer formation, whereas cells embedded in 3D Coll I appear as small cell clusters with numerous contacts to adjacent cells and interactions with their environment. We observed that HCT-116 and T47D formed steadily growing cell clusters, whereas A549 demonstrated the formation of loose cell structures (Figure 1B). Similar morphological transformations have been reported for various cells [10,11,13,15,24,39,40]. For instance, spherical growth was recently also described for A549 cells cultivated in Matrigel [24]. Glioma cell lines grew flat and spindle-like in 2D, whereas a round or ovoid shape was exhibited in Coll I scaffolds, thereby also forming clusters [13]. Human mesenchymal stem cells (MSCs) showed a more spherical morphology compared to 2D with distinct distribution patterns of focal adhesions [15]. Primary tendon cells in 3D exhibit a more tendon-like phenotype, which cannot be observed in 2D [11], and primary endothelial cells performed self-organization exclusively in 3D by forming capillaries [10]. All these observations suggest a more physiological cell morphology.

The morphological changes were accompanied by a markedly prolonged doubling time for all three cell lines; especially, T47D cells showed a more than 5-fold reduction in their proliferation, contrasting with standard 2D conditions (Figure 1C). Initially, HCT-116 showed a slight increase in cell number after three days within the Coll I matrix. For A549 and T47D, we were unable to detect an increase in cell numbers until day 3 or 5, respectively, implicating an adaptation phase after changing the cultivation system (Figure S2). Upon this adaptation phase, the cell number of all tested cell lines was continuously expanding over time. However, proliferation was highly reduced relative to the 2D standard cell culture (Figure 1C, Figures S2 and S3A,B). Reduced cell growth during 3D cultivation compared to plastic surfaces was already observed in previous studies [13,16,41,42]. In agreement with our study, Ashworth and colleagues determined a similar doubling time in a peptide-gel system for HCT-116 cells and demonstrated distinct growth profiles for each tested cell line [40]. The proportion of actively dividing cells might be reduced, as described for several pancreatic cancer cell line-derived spheroids, as well as for HaCaT cells in a Coll I-based spherical skin model [14,43,44], which could contribute to a decelerated proliferation rate. This finding is supported by studies in human colonic and mouse medulla cells, revealing a cell cycle distribution shift towards G1 upon 24 h in 3D [39,41]. In addition to cell cycle-associated factors, complex cellular adjustments such as the formation of contacts with surroundings, remodeling of the matrix architecture, and rearrangement of the cytoskeleton may contribute to the prolonged proliferation [4].

To ensure that the prolonged doubling time is caused by the new environmental conditions, and to exclude a higher mortality rate, cell viability was examined. A constant high viability rate throughout the 3D cultivation period could be verified for all cell lines (Figure 1D and Figure S3C). These data are consistent with previously described viability rates for MDA-MB-231, human mammary fibroblasts, and HaCaT cells cultured in Coll I-based 3D models [14,45,46]. The presence of consistently viable and steadily proliferating cells strongly indicates that this Coll I set-up is highly suitable for cell-based 3D applications.

In addition, we confirmed the non-invasive behavior for all three cell lines as previously described [47]. Invasion analysis revealed that HCT-116, A549 and T47D reached a maximum invasion distance of approximately 20 µm (Figure S4A) which was already described for the non-invasive cell line MCF-7 [48]. Moreover, none to only a few cells were determined as invasive cells below 20 µm (Figure S4B). In contrast to this, we used the known, highly invasive breast cancer cell line MDA-MB-231 as the positive control for invasion capability [45,47], which clearly demonstrated an invasive phenotype (max. invasive distance 75 µm, Figure S4A; 45% of cells below 20 µm, Figure S4B).

Taken together, we validated that Coll I-based 3D cultivation has a global effect on morphology, growth and proliferation of HCT-116, A549 and T47D, and is applicable to analyze cellular features such as invasion.

### 2.2. 3D Cell Culture Promotes Active Gene Transcription, Impacts the RNA Type Distribution and Induces Transcriptional Reprogramming That Affects Cellular Processes

**Figure 2:**
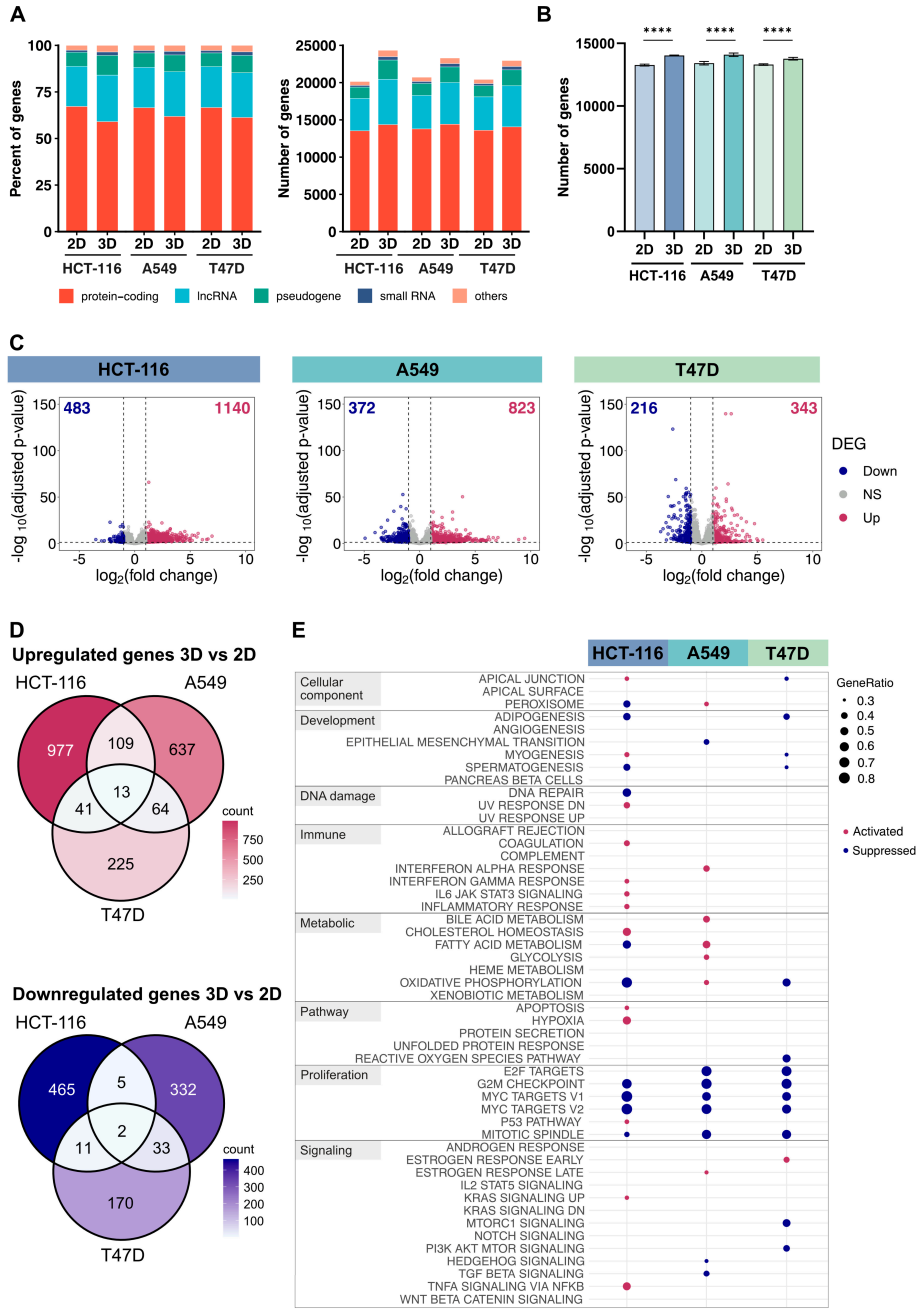
3D cultivation in Coll I matrices enlarges transcriptional complexity with impact on multiple key cellular processes. (**A**) Stacked bar plots representing the percentage as well as the number of genes in 2D and 3D for each gene biotype detected in RNA sequencing data set. The biotype annotation is based on the Ensembl database and collapsed into the shown groups with small RNA containing among others miRNA, snRNA, snoRNA and scaRNA and others, e.g., TEC, IG associated genes and rRNA. (**B**) Number of protein-coding genes in 2D and 3D. Data are shown as the mean values ± SD (*n = 3*). Statistical significance is indicated by asterisks (**** *p* < 0.0001). (**C**) Volcano plots of all protein-coding genes. Blue and dark red highlighted genes depicted as dots are significantly down- (*p*.adj. ≤ 0.05, log_2_(FC) ≤ −1) or upregulated (*p*.adj. ≤ 0.05, log_2_(FC) ≥ 1) during 3D compared to 2D. Thresholds are indicated by dashed lines. (**D**) VENN diagram of significantly up- (dark red) and downregulated protein-coding genes (blue) in 3D compared to 2D. (**E**) Enrichment plot of a GSEA (fold-change-based) on basis of the Hallmark pathway database. All Hallmark pathways with their associated category are shown with significantly enriched activated (red) and suppressed (blue) pathways (*p*.adj. ≤ 0.05) depicted as circles for each cell line comparing 3D to 2D. The Gene Ratio is included as size of the circles.

To investigate the observed phenotypical changes on transcriptomic level, we performed whole transcriptome sequencing. 3D-cultivated cells were compared to subconfluently grown cells in 2D. First, differences in RNA type distribution and number of expressed genes became apparent (Figure 2A). While the proportions of expressed protein-coding and non-coding genes were shifted to the non-coding species including lncRNAs, pseudogenes and small RNAs, the overall number of expressed genes increased. At least 2300 genes per cell line were additionally transcribed for the Coll I-embedded cells relative to their 2D counterparts (Figure 2B). These results demonstrate that the number of genes being actively transcribed is extended under 3D conditions and suggest that the transcriptome becomes more complex within the collagen matrix.

Upon quantitative analysis of the transcripts, we compared the RNA expression pattern between 2D and 3D cultivation for protein-coding genes to correlate it with the phenotypical changes. Hundreds of differentially expressed genes (DEGs) were identified, whereby HCT-116 showed the most comprehensive changes in their expression profile of 1623 DEGs compared to A549 and T47D with 1195 and 559 DEGs, respectively (Figure 2C). Interestingly, the majority of DEGs showed an upregulation pointing to an active response to the new environment by switching on specific gene expression. Similar results regarding the stimulation of gene expression could be shown for Matrigel- versus 2D-cultivated A549 cells [24].

The comparison of cell line-specific protein-coding DEGs uncovers only a few commonly upregulated and downregulated genes for all three cell lines (Figure 2D). This result suggests a highly individualized, cell line-specific response instead of a general response at the transcript level to the 3D environment. Commonly upregulated genes are associated with cell adhesion and metabolism. Among others, tubulin polymerization promoting protein family member 3 (*TPPP3*) and CREB3 regulatory factor (*CREBRF*) were promoted under 3D conditions. Both were already associated with cell cycle regulation in several cancer types [49,50,51,52,53].

Moreover, the gene set enrichment analysis (GSEA) based on the Hallmark gene sets revealed complex changes in various processes whereby immune-related processes were activated within the 3D cell culture, terms for cellular components, development, metabolic and signaling were differentially regulated, and proliferation-associated terms were mainly suppressed for all cell lines (Figure 2E). Previous studies described the accordance of transcriptomic changes and cellular phenotype upon 3D cultivation in A549, glioma cell lines as well as in human stem cells [11,13,15,18,24], supporting our initial observation. Moreover, an activation of immune-response-associated gene expression was proven for Matrigel-embedded A549 [24] cells and SiHa cell-derived spheroids [54]. These results reflect the morphological transformation of cancer cells, the reduced proliferative activity in 3D Coll I matrices, as seen before, and suggest an active cell–matrix interaction.

### 2.3. 3D-Induced Transcriptional Reprogramming of Cell Cycle, Energy Signaling and Cell–Matrix Interaction Occurs Gradually over Time

**Figure 3:**
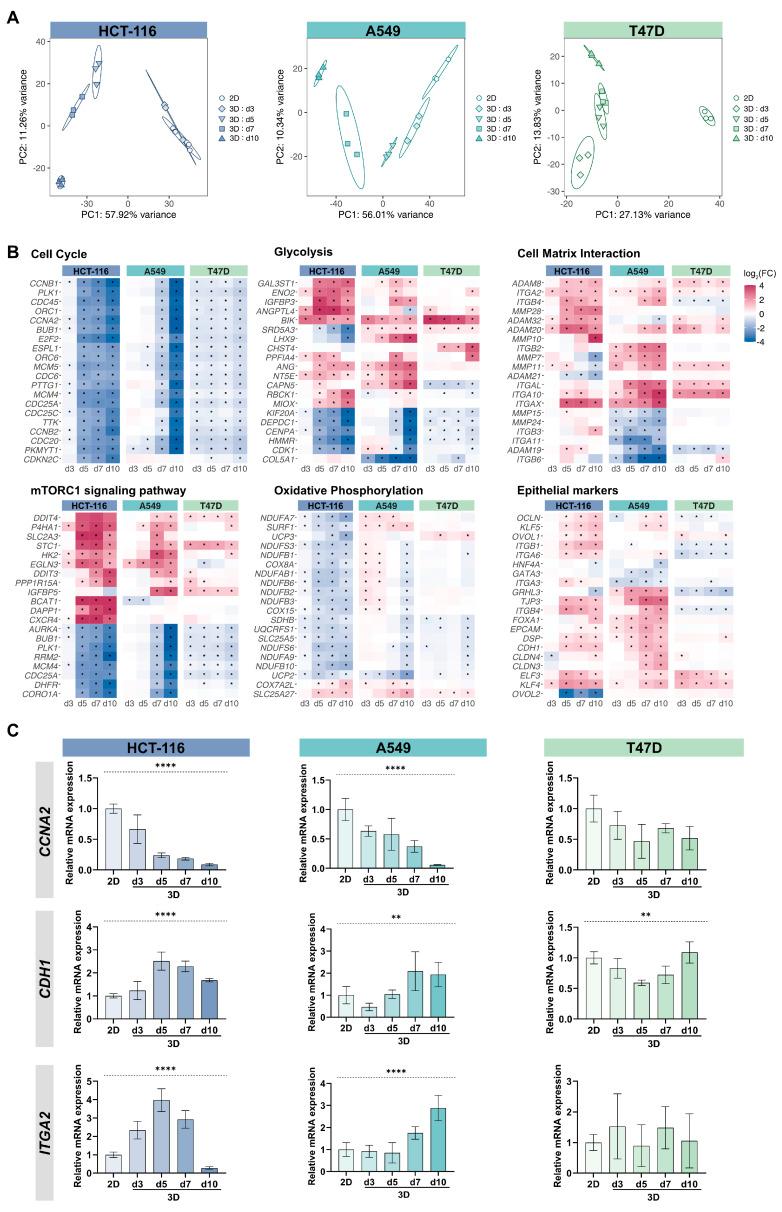
3D cultivation in Coll I matrices induces a gradual change in the gene expression over 10 days, especially in cellular processes such as cell cycle, mTORC1 signaling, and cell–matrix interaction. (**A**) PCA plots based on RNA sequencing data for all three cell lines comparing the gene expression of all protein-coding genes for cells cultivated in 2D and 3D for 3, 5, 7 and 10 days. (**B**) Heatmap of top 20 regulated genes in the gene clusters cell cycle, mTORC1 signaling, glycolysis (excluding mTORC1-related genes) and oxidative phosphorylation, cell–matrix interaction, and epithelial markers, showing the fold change after 3 to 10 days of 3D cultivation in comparison to 2D. Asterisks indicate an adjusted *p*-value below 0.05. (**C**) Relative *CCNA2*, *CDH1*, and *ITGA2* mRNA fold changes analyzed by RT-qPCR. Data are shown as mean ± SD (*n* = 3 to 4). Statistical significance was calculated using one-way ANOVA. The *p*-value is indicated by asterisks (* *p* < 0.05, ** *p* < 0.01, **** *p* < 0.0001) and describes a significant effect over time.

3D embedding may initiate a temporally ordered transcriptomic reprogramming with alterations across the already identified pathways. Therefore, we were interested in the temporal dynamics of gene expression during the 3D cultivation period and analyzed the gene expression pattern for 3 to 10 days for cells cultivated in Coll I matrices in comparison to 2D grown cells. The transcriptomic profile kept steadily changing over time in a cell line-specific manner (Figure 3A), supporting a progressive adaptation to the 3D environment. Gene expression patterns of components related to cellular processes such as cell cycle, mechanistic target of rapamycin complex 1 (mTORC1) signaling, glycolysis, oxidative phosphorylation, cell–matrix interaction, and epithelial markers were analyzed in more detail. Initially, we hypothesized significant alterations in gene expression already after 3 days in 3D relative to 2D. Surprisingly, RNA sequencing analysis revealed a notable degree of similarity between the transcriptomic profiles of both conditions. However, comparing the transcriptomic profile of 3D day 5 to day 10 with classical cell culture clearly demonstrated a transcriptional drift. In this drift, both up- and downregulated genes followed a unidirectional regulation pattern with maximum effect at day 7 or 10 upon embedding, except for some cell line-specific manifestations (Figure 3B).

Especially, all investigated cell cycle-related genes showed a straight and uniform reduction in their expression pattern (Figure 3B,C and Figure S5), which is in line with the increased doubling time for each cell line as well as the GSEA (Figure 2E). Interestingly, although HCT-116 displayed the highest growth ratio in 3D, the downregulation of cell cycle-associated genes was most pronounced compared to A549 and T47D. The reduction in cell cycle-related gene expression is also supported by a recently published study, where similar effects were observed for A549 cells cultivated in Matrigel for 5 days compared to 2D culture [24]. Next, genes essential for the mitochondrial respiratory chain were mainly downregulated during 3D cultivation (Figure 3B**),** whereas mTORC1 (Figure 3B and Figure S5) and glycolysis-related genes (Figure 3B and Figure S5) showed a more complex regulation. As a signal integration hub, mTORC1 regulates the cell growth, proliferation, metabolism and energy status of the cell in response to diverse external signals [55]. 3D embedded cells demonstrated a gradually decreasing gene expression for mTORC1 signaling promoters, such as budding uninhibited by benzimidazoles 1 (*BUB1*) and ribonucleotide reductase regulatory subunit M2 (*RRM2*), as well as mTORC1 signaling suppressing genes such as polo-like kinase 1 (*PLK1*) and murine double minute 4 (*MDM4*), whereas mTORC1 inhibitor DNA damage-inducible transcript 4 (*DDIT4*) was strongly activated upon day 5. Similar results were revealed for glycolysis-associated components. While genes encoding for key enzymes, e.g., hexokinase 2 (*HK2*) and enolase 2 (*ENO2*), were upregulated over time, genes related to the Warburg effect (*DEPDC1*, *AURKA*, *CENPA* and *HMMR1*) got repressed. These data strongly point to a differential reprogramming of the cellular energy metabolism with 3D cultivation (Figure 3B).

The addition of Coll I-based microenvironment represents a tremendous change for the cell by adding complex mechanical stimuli and nutrient supply alterations. This adaptation can be followed by the differential regulation of mTORC1 signaling. Similarly, key enzymes of the carbohydrate metabolism as well as the PI3K-AKT-mTOR pathway were reported to be downregulated in different 3D set-ups [39,41]. Among others, a mTORC1 activation by mechanical signals was proposed for skeletal muscle cells [56]. Thus, Coll I-induced mechanical forces might stimulate mTORC1 signaling as well in our system. Proven evidence exists for mTORC1-mediated cell cycle regulation. Recently, a study with Hela and U2OS cells highlighted the tight connection of mTORC1 activity and the progression through cell cycle phases [57]. Moreover, the bidirectional mTORC1 regulator PLK1 does also positively regulate cell cycle progression [58,59]. Hence, suppression of *PLK1* and cell cycle-associated genes such as *cyclin B1* (*CCNB1)* and *CCNB2*, which are essential in the early stages of mitosis [60,61], might contribute together to delayed cell cycle progression.

Next to the downregulation of cell cycle components, genes associated with oxidative phosphorylation and glycolysis, two fundamental pathways linked with the energy status of the cell, were also found to be either repressed or differentially regulated, respectively. Unlike traditional cell cultivation, the cell culture medium diffuses into the Coll I fibril network surrounding the cells. The regulation of both mentioned pathways might be a response to the limited nutrient supply and indicates a switch in the cell metabolic state. As mentioned above, *HK2* and *ENO2* were upregulated in 3D for all three cell lines for the analyzed time period. An upregulation for both was also demonstrated for MCF7, which was accompanied by an elevated glucose consumption [62]. Similar effects were reported for tumor-on-chip models. Here, U251-MG and A549 showed a difference in consumption of glucose or glutamine and lactate production. These metabolic changes were associated with a switch from oxidative to glycolytic phenotype [42]. In 3D microenvironments, gradients of soluble factors that emerge from various diffusion-modulating structural features, as well as pressure gradients and matrix deformations, are present. Nevertheless, comparing the metabolic state of cells grown in different systems revealed that 3D cultured cells were more comparable to original tissues than 2D cultures [41].

In contrast to this, modulators of cell–cell and cell–matrix interaction including diverse integrins, a disintegrin and metalloprotease (*ADAM*) family members, as well as epithelial markers, were mainly upregulated compared to 2D conditions (Figure 3B,C and Figure S5). This increased expression further supports the cell–matrix interaction in our setup, suggesting an active ECM remodeling for progressive cell growth and expansion, which is also in line with our observations (Figure S2). Similarly, functional enrichment analysis of A549 and BEAS-2B cells grown in Matrigel revealed a promotion of cell adhesion and cell–matrix interactions, respectively [24]. Integrins, as transmembrane receptors, maintain adhesion via direct connection of the cytoskeleton with the surrounding ECM. The differential regulation of integrins and cyclins correlated with reduced cell proliferation, similar to reports for breast cancer cells in a 3D environment [63,64] supporting our data. Moreover, the EMT marker E-cadherin (*CDH1*) was observed to be upregulated in HCT-116 and A549 over time in 3D. *CDH1* was also described as a non-invasive marker [65,66]. In contrast, known invasion markers such as NADH:ubiquinone oxidoreductase subunit A8 (*NDUFA8)*, Sjögren’s syndrome nuclear autoantigen 1 (*SSNA1*) [67] and catenin β1 (*CTNNB1*) [65,68] were downregulated (Figure S6), underlining the non-invasive phenotype.

Interestingly, several components of cell–matrix interactions, epithelial markers, and oxidative phosphorylation initially displayed a different regulation between HCT-116 and the two other cell lines when comparing 2D to 3D. However, the shift in gene regulation towards day 10 in 3D shows often a similar tendency as for HCT-116. A completely individual regulation was demonstrated for several matrix metalloproteases (MMPs) such as *MMP7*, *MMP11*, *MMP15* and *MMP24* (Figure 3B). Here, each cell line seemed to promote specific MMPs to actively interact with the Coll I matrix.

Next to important cellular processes, we were also interested in a more practical aspect: the impact of the surrounding matrix on well-known housekeeping genes. We assessed the gene expression by WTS and RT-qPCR for glyceraldehyde-3-phosphate dehydrogenase (*GAPDH*), beta-actin (*ACTB)*, TATA-box binding protein (*TBP*), and the spliceosome-associated protein CWC15 homolog (*CWC15*). Surprisingly, the comparative data analysis revealed an unexpected challenge for *ACTB* and *GAPDH.* These housekeepers exhibited varying expression patterns under 3D conditions. However, *TBP* and *CWC15* demonstrated more stable expression values within our RNA sequencing data set and were proven to be more stable across all 2D and 3D conditions (Figures S7 and S8). *CWC15* was previously described for HCT-116 under classical cell culture conditions [69]. Especially, *GAPDH* showed a strong fluctuation in transcript level during the culturing period in Coll I matrix. In line with this, GAPDH protein was shown to accumulate after 2 days in LS174T, HT29 and MCF7 spheroids [39]. In contrast, HepaRG cells displayed similar RNA expression levels for *GAPDH* under 2D and 3D [70], indicating different regulations of GAPDH in the stated cell lines under 3D conditions. In summary, the stability of reference genes should always be evaluated under all experimental conditions to ensure reliable results.

### 2.4. Substantial Induction of lncRNA Transcription Is Promoted by Coll I Microenvironment

**Figure 4:**
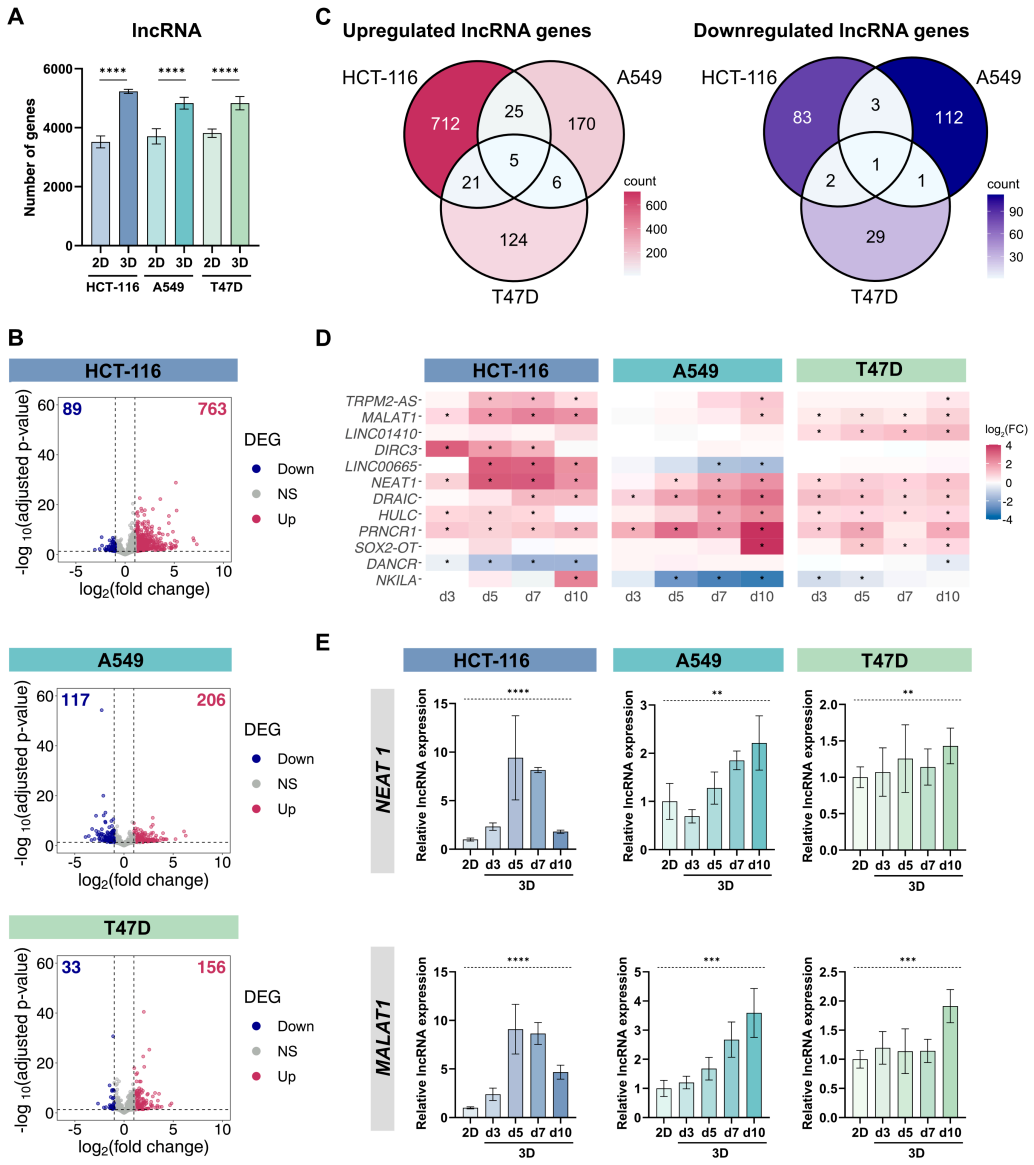
Number of transcribed lncRNAs massively increased and differentially expressed lncRNAs were mainly upregulated under 3D conditions. (**A**) Number of lncRNA-encoding genes in 2D and 3D for all three cell lines detected by RNA sequencing. Data are shown as the mean values ± SD (*n = 3*). The *p*-values are indicated as asterisks (* *p* < 0.05, ** *p* < 0.01, *** *p* < 0.001, **** *p* < 0.0001). (**B**) Volcano plots of all lncRNA-coding genes depicted as dots. Blue and dark red highlighted lncRNA-coding genes are significantly down- (*p*.adj. ≤ 0.05, log_2_(FC) ≤ −1) or upregulated (*p*.adj. ≤ 0.05, log_2_(FC) ≥ 1) during 3D cultivation in Coll I matrices compared to 2D cultivation. Thresholds are indicated by dashed lines. (**C**) VENN diagram of significantly up- (dark red) and downregulated (blue) lncRNA genes in 3D compared to 2D. (**D**) Heatmap of cancer-related lncRNA genes showing the fold regulation after 3 to 10 days of 3D cultivation in comparison to 2D. Asterisks indicate an adjusted *p*-value below 0.05. (**E**) Relative *NEAT1* and *MALAT1* lncRNA fold change analyzed by RT-qPCR. Data are shown as mean ± SD (*n =* 3). Statistical significance was calculated using one-way ANOVA. The *p*-value is indicated as asterisks (* *p* < 0.05, ** *p* < 0.01, *** *p* < 0.001, **** *p* < 0.0001) and describes a significant effect over time.

LncRNAs have increasingly been coming to the forefront of cancer research. During our bioinformatic analysis, we noticed a remarkable increase in the number of expressed lncRNA encoding genes by 26.6–48.7% when cells are embedded in Coll I matrices (Figure 4A), which was even more pronounced as observed for the protein-coding genes. Thus, the number of actively transcribed lncRNA-coding genes increased to a higher extent and its proportion expanded relative to protein-coding genes under 3D conditions. Both the induction of protein- and lncRNA-coding genes highly points to a more complex RNA profile of all tested cell lines in response to the new cellular environment. For differentially regulated lncRNA genes, the majority of lncRNAs got promoted, whereas only a small proportion was downregulated compared to 2D (Figure 4B). However, most lncRNAs displayed a cell line-specific expression pattern for up- as well as downregulated genes whereby only six commonly regulated lncRNAs were identified (Figure 4C). This suggests more individualized regulation, similarly to our observations regarding protein-coding DEGs (Figure 2D). Next, we selected known cancer-related lncRNAs [29] for further analysis of temporal expression dynamics in 3D-cultivated cells from day 3 to day 10 compared to 2D cultivation (Figure 4D,E and Figure S9; Table S1). The majority of these genes showed an increased expression under 3D conditions, including *DRAIC*, *PRNCR1*, *HULC*, *SOX2-OT*, *TRPM2-AS*, *DIRC3*, *NEAT1*, *MALAT1* and *LINC01410*, whereas *DANCR*, *LINC00665* and *NKILA* were differently regulated among the analyzed cell lines. Temporal dynamics could be observed for almost all genes, which were also validated for *NEAT1*, *MALAT1*, *TRPM2-AS* and *DANCR* via RT-qPCR (Figure 4E and Figure S9). These four lncRNAs are known to encourage cancer progression of several cancer types in various manners as they are described to promote proliferation and associated with a poor prognosis for patients [71,72,73,74,75,76,77,78,79,80,81,82]. Their tumor progressive relevance is, moreover, reflected in their role in contributing to invasiveness [71,72,73,75,76,77,78,79,80,81,82,83], but also, the opposite was described in previous studies for *NEAT1* and *MALAT1* [84,85,86,87,88]. Mechanistically, most of these studies describe miRNA-sponging and gene/pathway regulation. *NEAT1* is used to form paraspeckles, membraneless nuclear bodies. These subnuclear structures trap specific RNAs and represent cellular buffer systems in order to rapidly alter gene expression [89].

Further, these lncRNAs are of high relevance in metabolic reprogramming [71,90,91,92,93,94,95], affecting primarily glucose metabolism [71,90,91,93]. For *DANCR*, a further influence on fatty acid synthesis is described in triple-negative breast cancer [94]. LncRNAs were also shown to influence cell–matrix interactions by affecting matrix remodeling [81,96,97]. *MALAT1* has been described to modify the tumor microenvironment facilitating cell proliferation and metastasis [98], and observed to affect cell–matrix interactions in endothelial cells [99]. The surrounding matrix can in turn affect lncRNA-mediated cellular functions: *NEAT1*-associated paraspeckle integrity [100] is mechanosensitive. In several cancer cell lines, an increase in paraspeckles with decreasing matrix stiffness was described [101]. Further described cancer-related functions of lncRNAs include modulation of therapy resistance, immune response, and angiogenesis for diverse origins [29]. With this, our data point to a widespread reprogramming for various RNA types not only for genes encoding proteins, but also for regulatory lncRNAs resulting in effects on the most important cancer-related processes.

Elevated expression levels of lncRNAs have previously been reported in spheroids, which is consistent with our observations in 3D. Here, increased transcription levels of *MALAT1* and *NEAT1* were determined in spheroids formed from Huh7 and HepG2 cells, being important for spheroid formation, and have been positively associated with cancer stem cell properties [102,103]. The expression of lncRNA *CYTOR* was found to be inversely correlated with, and therefore dependent on, matrix stiffness in human MSCs [104]. Interestingly, several studies investigating the gene expression pattern of cancer patient-derived samples relative to healthy tissue reported an upregulation of lncRNAs within the tumor sample. For instance, increased expression levels were demonstrated in patient samples for *DRAIC/lnc152* in renal carcinoma [105] and breast cancer [106]; for *PRNCR1* in colorectal cancer [107], non-small cell lung cancer [108] and breast cancer [109]; and for *HULC* [110] and *MALAT1* [102] in liver cancer. These data strongly suggest that results obtained from 3D cell cultivation better reflect tumor nature.

## 3. Materials and Methods

### 3.1. Cell Culture in 2D and 3D

The human cell lines HCT-116 (colon carcinoma, ECACC 91091005), A549 (non-small cell lung cancer, ECACC 86012804), T47D (breast carcinoma, DSMZ ACC 739), and MDA-MB-231 (breast carcinoma, DSMZ ACC 732) were cultivated as recommended in McCoy’s 5A, F-12K, RPMI and Leibovitz L-15, respectively. Each medium was supplemented with 10% fetal bovine serum (FBS) and 1% Penicillin/Streptomycin. Cell cultivation and all incubation steps were performed at 37 °C, 5% CO_2_ and 95% humidity. MDA-MB-231 were cultivated at 0% CO_2_. Unless otherwise stated, cell culture medium and consumables were obtained from Life Technologies (Darmstadt, Germany). Cell lines were regularly tested negative for mycoplasma contamination by PCR (Takara Bio, Saint-Germain-en-Laye, France; Transgen Biotech, Beijing, China) and authentication was confirmed by STR analysis (Eurofins, Ebersberg, Germany).

For 2D culture, cells were seeded in TC-treated culture flasks (Corning, Amsterdam, The Netherlands) and grown until reaching 70 to 90% confluence. Cells were washed twice with pre-warmed Dulbecco’s phosphate-buffered saline (DPBS, Capricorn, Ebsdorfergrund, Germany) and detached using 0.25% Trypsin-EDTA solution for 5 min at 37 °C. Digestion was stopped with 4 volumes of complete medium followed by centrifugation at 264× *g* for 5 min at room temperature.

For the analysis of Coll I fibrillogenesis, 100 µL of different concentrations of rat tail collagen type I (Corning) in 250 mM phosphate buffer (pH 7.5) were transferred to a pre-cooled 96-well microplate (Corning). Coll I polymerization at 37 °C was monitored in a microplate reader (TECAN Spark, TECAN, Crailsheim, Germany) by measurement of turbidity at 405 nm for 60 min with intervals of 1 min. The measurements were performed in four independent experiments.

For the cultivation of single cells embedded in Coll I matrices, cells were carefully resuspended in 250 mM phosphate buffer (pH 7.5) and mixed with Coll I at 4 °C to a final concentration of 2.5 mg/mL. The seeding cell number, which was kept constant across the entire experimental setup for each cell line, is stated in Table S2. The total volume of 300 µL were transferred to an ibitreat 24-well plate (ibidi), and fibrillogenesis of Coll I was enabled at 37 °C, 5% CO_2_ for 50 min to homogeneously embed cells. Subsequently, Coll I matrices were washed twice with pre-warmed DPBS, and 1 mL cell culture medium was added. Cells were cultivated for the indicated time points.

### 3.2. Determination of Cell Number and Viability of 2D- and 3D-Cultivated Cells

To determine cell number and viability, 3D-cultivated cells were released enzymatically. To achieve this, Coll I matrices were transferred to an empty well, washed twice with DPBS and subsequently incubated with 1000 U/mL collagenase IV (Thermo Fisher Scientific, Darmstadt, Germany) at 37 °C for 20 min. Digested hydrogels were merged with one volume of complete medium. Cells were pelleted at 264× *g* for 5 min and resuspended in DPBS. 2D-cultivated cells were counted upon centrifugation and resuspension. Cells were analyzed using Countess 3 FL Automated Cell Counter (Thermo Fisher Scientific) according to the manufacturer’s protocol. Cellular parameters were analyzed in at least three independent experiments. To calculate growth ratios, cell count was normalized to the seeding cell count, which was determined for 3D cell culture by analyzing separate aliquots of cells resuspended in 250 mM phosphate buffer (pH 7.5).

### 3.3. Cell Invasion Analysis

For invasion analyses, Coll I matrices were prepared on glutaraldehyde (Roth, Karlsruhe, Germany; 0.5%)-coated glass coverslips (VWR; 13 mm diameter), allowing covalent attachment of Coll I via lysine residues [111]. Coll I matrices were reconstituted by mixing collagen I with 250 mM phosphate buffer (pH 7.5) at 4 °C to a final concentration of 2.5 mg/mL. Volumes of 40 µL Coll I solution were transferred to glass coverslips, and fibrillogenesis was enabled at 37 °C, 5% CO_2_ for 50 min resulting in matrices of approx. 300 µm thickness. Prior to cell seeding, matrices were washed twice with pre-warmed DPBS and equilibrated with the appropriate cell culture medium for 1 h at 37 °C. Defined cell numbers were seeded per coverslip and cultivated for four days (Table S2). For fluorescence microscopy, cells were washed three times with DPBS and fixed in 4% formaldehyde (Sigma-Aldrich/Merck, Taufkirchen, Germany) for 15 min at room temperature. Cells were washed three times with DPBS, permeabilized using 0.1% Triton X-100 (Roth) for 15 min, and stained with 1 µg/mL DAPI (Roth) for 20 min (all steps at room temperature). Cells were washed three times with DPBS and imaged using a Nikon Ti2-E system (Nikon, Amsterdam, The Netherlands) with a 10× objective (NA 0.3). Images were taken in DAPI-channel in steps of 5 µm distance for a total depth of 100 µm. Cellular localizations were analyzed in a defined region of interest (ROI) of 750 × 1000 pixel (approx. 550 × 734 µm) by DAPI signal (three coverslips, two positions each). At least three independent experiments were performed per cell line. The Z-stack fluorescent image series was analyzed using a positive cell detection algorithm in QuPath (v0.6.0), based on DAPI fluorescence. Cell detections were filtered according to morphology and intensity-based criteria and assigned to a Z-level based on maximal nuclear sharpness. Invasiveness was quantified as the percentage of cells located below 20 µm. Maximal invasion depth was defined as the mean of the deepest 10% of cells.

### 3.4. RNA Isolation, cDNA Synthesis and Gene Expression Analysis

RNA was isolated from 2D-cultivated cells at 70% confluency and from 3D-cultivated cells on day 3, 5, 7 and 10.

Cells grown in cell culture flasks or Coll I matrices were washed twice with DPBS. Cells were lysed and matrices were dissolved using 1 mL QIAzol Reagent (Qiagen, Hilden, Germany). To these, 200 µL chloroform (Roth) per mL QIAzol were added, followed by homogenization of the suspension and centrifugation at 4 °C, 12,000× *g* for 15 min. The aqueous phase was merged with 1 volume cold isopropanol (Roth) and RNA was precipitated at -20 °C for 3 days. Precipitated RNA was washed three times with 75% ethanol (ITW Reagents, Monza, Italy) and dissolved in RNase-free H_2_O. For each cell line, at least three independent experiments were performed.

For each sample, 1 µg isolated RNA was transcribed to cDNA by using PrimeScript™ RT Reagent Kit (TakaraBio, Tokyo, Japan) with gDNA Eraser (Takara), as described by the manufacturer. To obtain cDNA used for lncRNA expression analysis, 0.5 µg isolated RNA was transcribed through the application of RT^2^ First Strand Kit (Qiagen) according to manufacturer instructions. Reverse transcription reaction was carried out at 37 °C for 60 min.

Expression of selected genes was determined by RT-qPCR. cDNA was mixed with iTaq Universal SYBR Green Supermix (Bio-Rad, Feldkirchen, Germany) and specific primers (Table S3) according to the manufacturer’s protocol. The 2-step RT-qPCR with melting curve analysis was performed with the C1000 Touch Thermal Cycler including the CFX96 Optics module (Bio-Rad). Data were analyzed by using the relative standard curve method. To perform expression analysis of lncRNAs, *NEAT1* (GeneGlobe ID: LPH15809A-200), *MALAT1* (GeneGlobe ID: LPH18065A-200), *DANCR* (GeneGlobe ID: LPH06287A-200) and *TRPM2-AS* (GeneGlobe ID: LPH08274A-200), and the internal control *CWC15* (GeneGlobe ID: PPH09182A-200), the RT^2^ (lncRNA) RT-qPCR Primer Assays (Qiagen), and the RT^2^ SYBR Green RT-qPCR Mastermix (Qiagen) were used according to manufacturer instructions.

### 3.5. Whole Transcriptome Sequencing and Bioinformatic Analysis

Library preparation, sequencing, demultiplexing, and adapter trimming were performed by CeGaT GmbH (Tübingen, Germany). Sequencing libraries were generated using the KAPA RNA HyperPrep Kit with RiboErase (HMR) in combination with KAPA Globin Depletion Hybridization Oligos (Roche, Basel, Switzerland), following the manufacturer’s protocol. For most samples, 100 ng of total RNA was used as input. Four samples contained <59 ng RNA and were processed with the available amount. Libraries were sequenced on an Illumina NovaSeq 6000 system in 101 bp paired-end mode, yielding between 32 and 53 million paired-end reads per sample with Q30 values ≥ 91.33% across samples. Demultiplexing was performed with bcl2fastq v2.20, and adapter trimming was carried out using Skewer v0.2.2 [112].

Downstream bioinformatic analyses were conducted using an in-house Nextflow v25.04.7 [113] pipeline. Reads were aligned to the human reference genome GRCh38 (Ensembl release 110) using HISAT2 v2.2.1 [114] with the parameter --rna-strandness FR. Resulting BAM files were sorted and indexed with SAMtools v1.20 [115]. Transcript-level quantification was performed using HTSeq-count v2.0.9 with the following parameters, -s yes -t exon -m intersection-strict --nonunique=fraction, using the corresponding GRCh38 (Ensembl release 110) GTF annotation [116].

All analyses were performed in R (v4.5.2) [117]. Raw transcript-level counts were aggregated to gene-level counts by summation. Genes with fewer than 1 count across all samples were removed prior to downstream analysis. For visualization, variance-stabilizing transformations were applied using the regularized log (rlog) function from DESeq2 v1.48.1 [118]. Sample relationships were assessed for two independent sets of HCT-116 samples (each n = 3). They showed a Pearson correlation coefficient of R ≈ 1.0 and were merged into a single group (n = 6) for all following differential expression analyses.

Differential expression analysis was carried out using DESeq2, including size-factor normalization and dispersion estimation. Wald tests with apeglm shrinkage of log_2_ fold changes were applied, and significance was determined using Benjamini–Hochberg-adjusted *p*-values [119]. Genes were considered significantly differentially expressed if they exhibited an absolute log_2_ fold change ≥ 1 and an adjusted *p*-value ≤ 0.05.

Uniquely expressed genes were identified by selecting genes with expression >0.3 TPM in at least one sample of one condition, while all samples in the other condition had <0.3 TPM.

Gene biotypes were obtained from the Ensembl database and collapsed for visualization into protein-coding, lncRNA, pseudogene (all biotypes containing the term pseudogene), small RNA (miRNA, scRNA, snRNA, scaRNA, snoRNA), and others (all remaining biotypes).

Enriched biological pathways were identified by using the gene set enrichment analysis package fgsea v1.34.2 [120] based on the hallmark gene sets by the Molecular Signatures Database (MSigDB) [121,122].

### 3.6. Statistical Analysis

All data depicted as bar plots are presented as mean ± standard deviation (SD). Data shown as whisker-box plots are presented as a box spanning the interquartile range (25th to 75th percentile) with whiskers extending from the minimum to the maximum. Growth ratios and number of protein-coding and lncRNA genes were analyzed using one-way ANOVA with subsequent Tukey’s post hoc test for multiple comparison correction. For comparing two groups regarding invasion distance and percentage of invasive cells, unpaired two-tailed Student’s *t*-tests were performed. For comparing the time course regarding relative mRNA expression, one-way ANOVA was conducted. These analyses were performed using GraphPad Prism software (v10; GraphPad Software Inc., La Jolla, CA, USA). Statistical differences were considered for *p*-values below 0.05 and are depicted in the plots as stars as stated in the figure description.

## 4. Conclusions

To our knowledge, this is the first direct side-by-side comparison of the non-invasive cancer cell lines HCT-116, A549, and T47D cultured in a 3D Coll I microenvironment compared with traditional 2D cultivation in a time-dependent manner. Thereby, this study demonstrates comprehensive reprogramming under 3D culture conditions on transcriptome level which is accompanied by a phenotypic adaptation. In addition to a predominant upregulation of protein-coding genes and long non-coding transcripts, we uncovered a more complex transcriptomic landscape for cells embedded in Coll I matrices as a direct response to the new environment. Utilizing Coll I 3D matrices appears to be a valuable tool to characterize cancer cell behavior under conditions that mimic a physiological environment more closely for downstream applications focusing on clinically relevant factors, such as deciphering tumorigenic interaction networks, evaluating biomarkers, and developing anti-cancer drugs.

## Data Availability

The transcriptomic data sets are available in the BioStudies database (https://www.ebi.ac.uk/biostudies, accessed on 31 July 2026), under accession number E-MTAB-16838.

## Supplementary Materials

The following supporting information can be downloaded at: https://www.mdpi.com/article/10.3390/ijms27156907/s1.
