# Supplementary material for: Adaptation of Non-Invasive Cancer Cells to 3D Collagen I Microenvironment Induces Transcriptional Reprogramming Accompanied by a More Complex RNA Landscape
Source: Int J Mol Sci. 2026 Aug 1;27(15):6907. doi: 10.3390/ijms27156907 (PMC13467458; doi:10.3390/ijms27156907)
Supplement: Supplementary file 1 [file ijms-27-06907-s001.zip › ijms-4309508-supplementary.pdf]

Supplementary Information

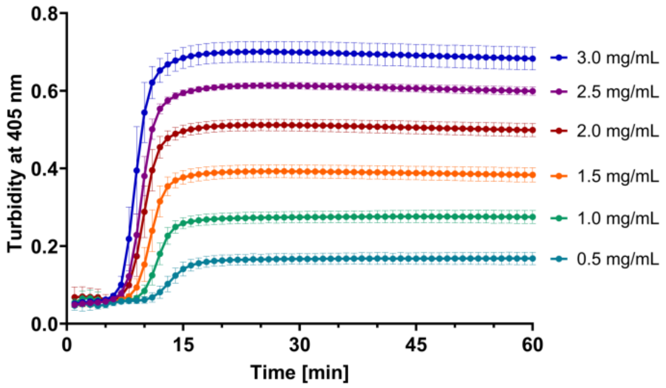

Figure S1: Analysis of fibrillation kinetics in dependence on Coll I concentration.
Increase in turbidity at 405 nm is shown during the process of fibrillogenesis. Data of four independent experiments are shown as mean values ± SD.

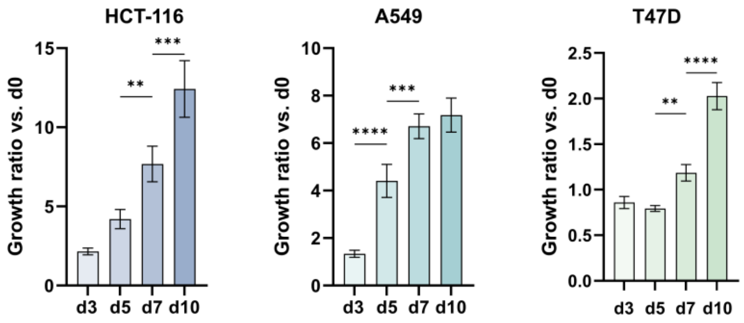

Figure S2: Growth ratios in 3D.
Cellular growth ratios of 3D cultivated cells relative to day 0 for indicated time points (n = 4). Data are shown as mean values ± SD. Statistical significance was calculated using one-way ANOVA with subsequent Tukey's post hoc test for multiple comparison correction. The p-value is depicted as stars (* p < 0.05, ** p < 0.01, *** p < 0.001, **** p < 0.0001).

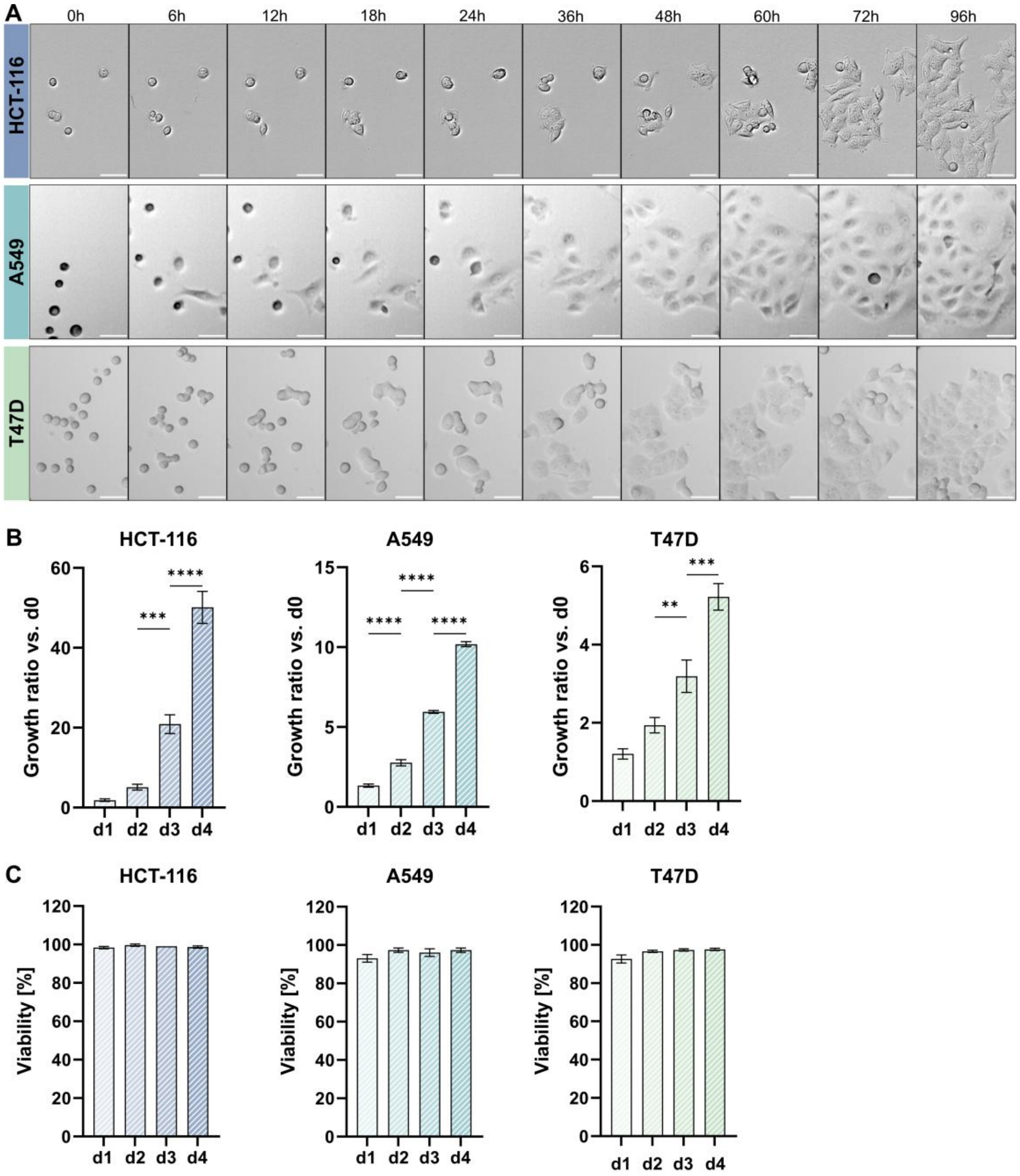

Figure S3: Cellular growth parameters in 2D.

A. Representative bright-field images of HCT-116, A549 and T47D cells cultivated in 2D for indicated time periods (Scale bar: 50 µm). **B.** Cellular growth ratios of 2D cultivated cells relative to day 0 for indicated time points (*n* = 3). Statistical significance was calculated using one-way ANOVA with subsequent Tukey's post hoc test for multiple comparison correction. The p-value is depicted as stars (* $p < 0.05$, ** $p < 0.01$, *** $p < 0.001$, **** $p < 0.0001$). **C.** Viability analysis of 2D cultivated cells at indicated time points (*n* = 3). Cell counts as well as viability values were determined by automated bright-field image analysis. Data are shown as mean values ± SD.

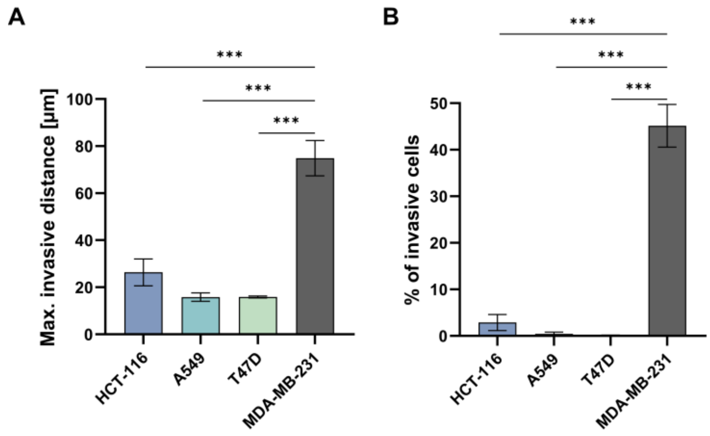

Figure S4: Analysis of cell invasion in 3D Coll I matrices.
A. Maximal invasion depth of HCT-116, A549, T47D and MDA-MB-231 (*n* = 3). **B.** Percentage of invasive cells for HCT-116, A549, T47D, and MDA-MB-231 (*n* = 3). Data are shown as mean values ± SD. The p-value is depicted as stars (* $p < 0.05$, ** $p < 0.01$, *** $p < 0.001$, **** $p < 0.0001$).

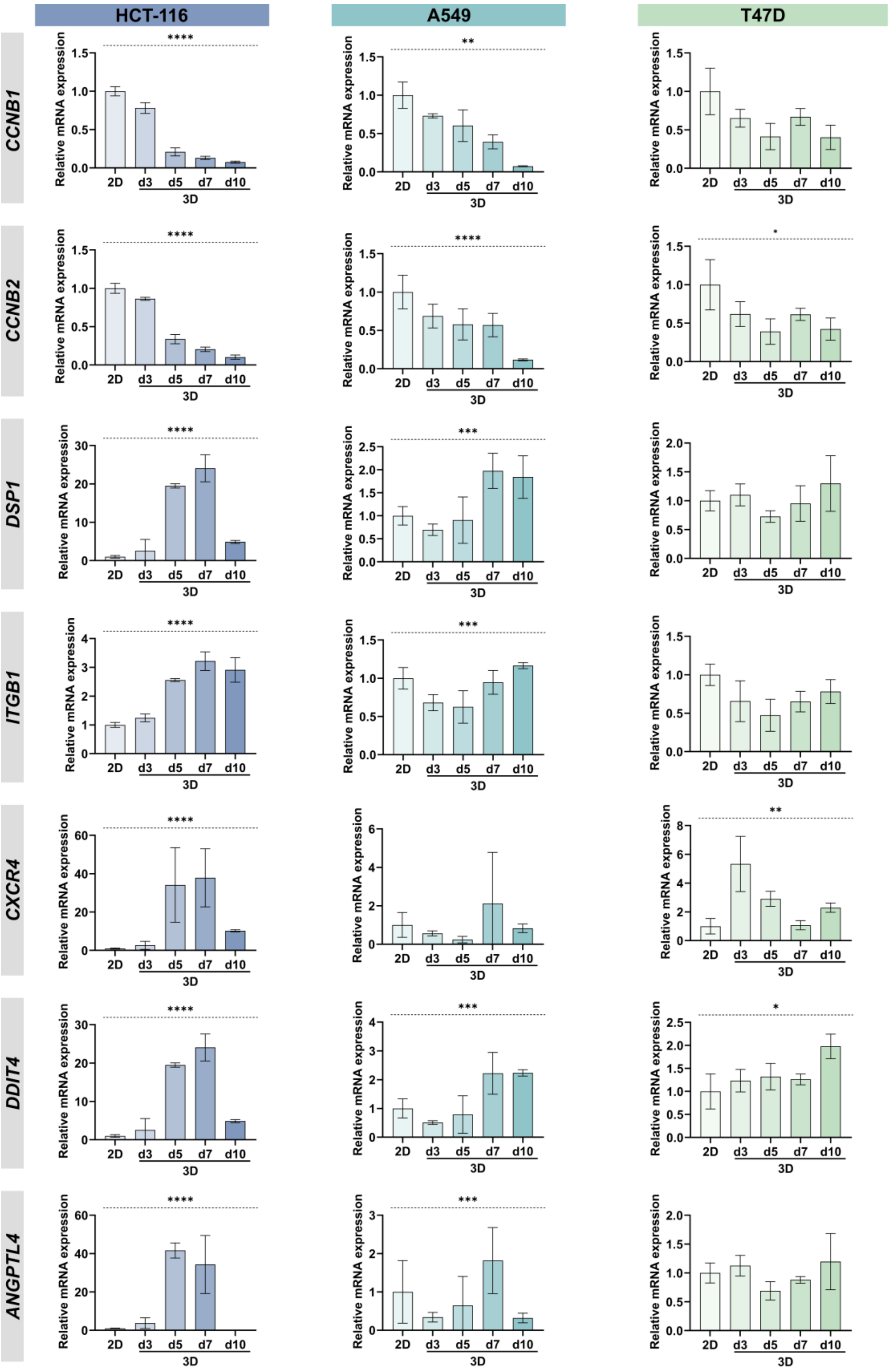

Figure S5: Analysis of relative expression of key regulated protein-coding genes via RT-qPCR.
Relative mRNA expression of *CCNB1*, *CCNB2*, *DSP1*, *ITGB1*, *CXCR4*, *DDIT4*, and *ANGPTL4* normalized to *CWC15* and 2D for all three cell lines determined by RT-qPCR (n = 3 to 4). Data are shown as mean values ± SD. Statistical significance was calculated using one-way ANOVA. The p-value is depicted as stars (* $p < 0.05$, ** $p < 0.01$, *** $p < 0.001$, **** $p < 0.0001$) and describes a significant effect over time.

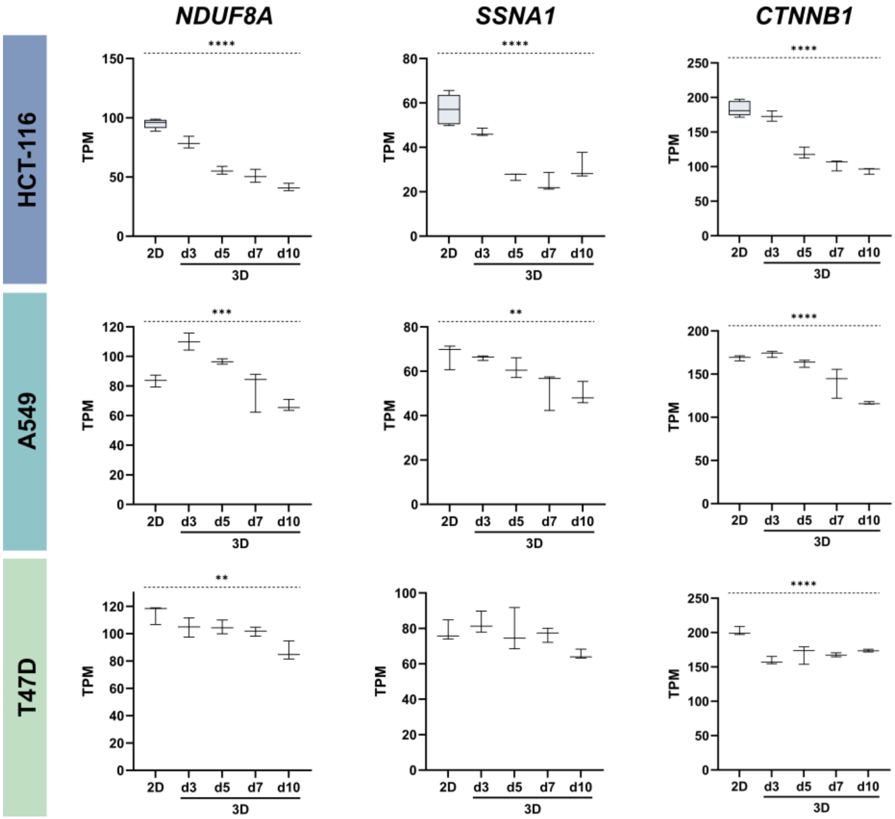

Figure S6: Gene expression of known invasion-associated markers.
TPMs of the invasion-associated marker genes *NDUF8A*, *SSNA1*, and *CTNNB1* for all three cell lines (*n* = 3). Data are shown as whisker-box plots with minimum and maximum. Statistical significance was calculated using one-way ANOVA. The p-value is depicted as stars (* $p < 0.05$, ** $p < 0.01$, *** $p < 0.001$, **** $p < 0.0001$) and describes a significant effect over time.

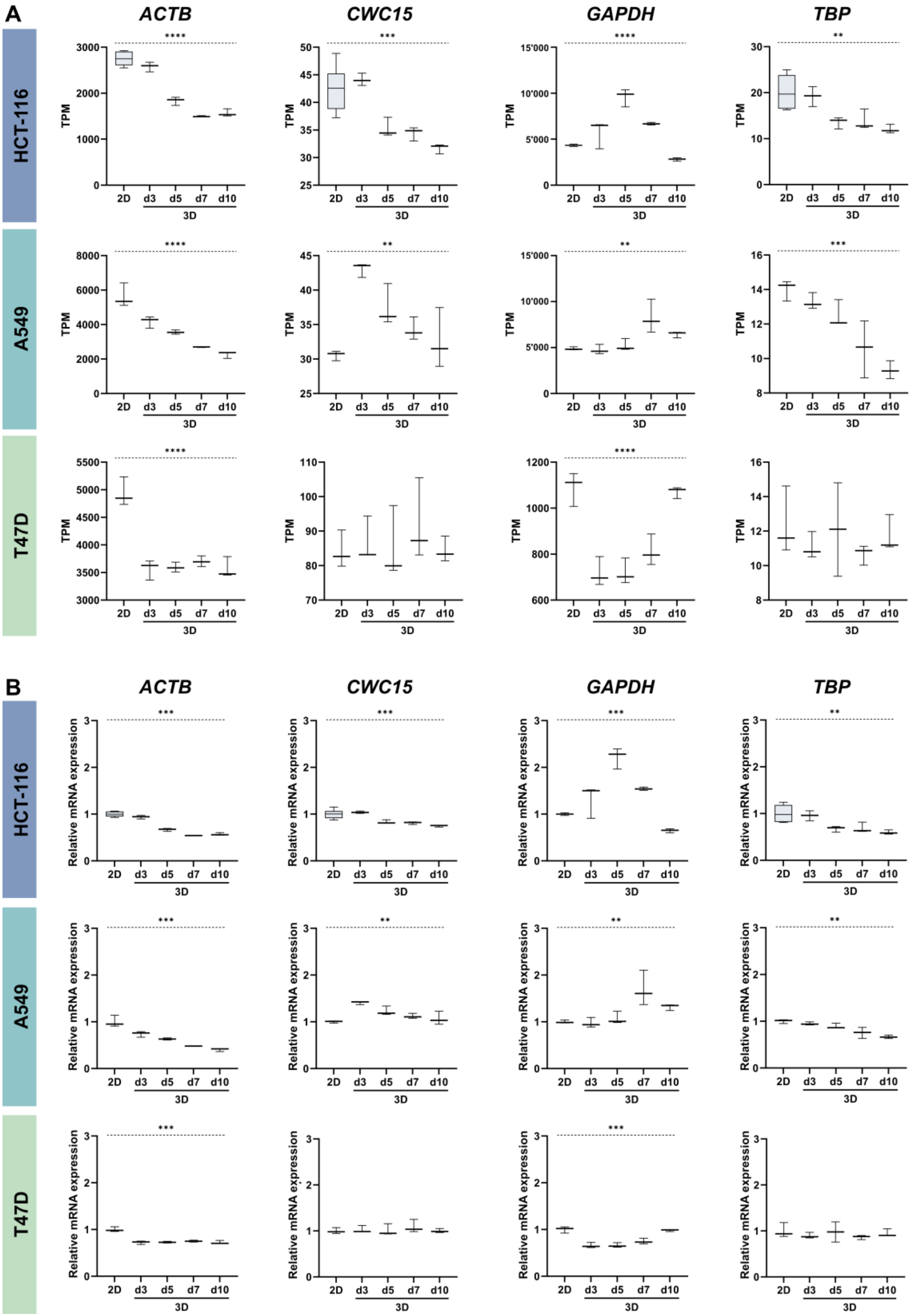

Figure S7: Comparison of housekeeper gene expression analyzed via RNAseq.
A. TPMs of the housekeeper genes *ACTB*, *CWC15*, *GAPDH*, and *TBP* for all three cell lines (n = 3). **B.** Relative mRNA expression of *ACTB*, *CWC15*, *GAPDH* and *TBP* normalized to 2D for all three cell lines. Data are shown as whisker-box plots with minimum and maximum. Statistical significance was calculated using one-way ANOVA. The p-value is depicted as stars (* $p < 0.05$, ** $p < 0.01$, *** $p < 0.001$, **** $p < 0.0001$) and describes a significant effect over time.

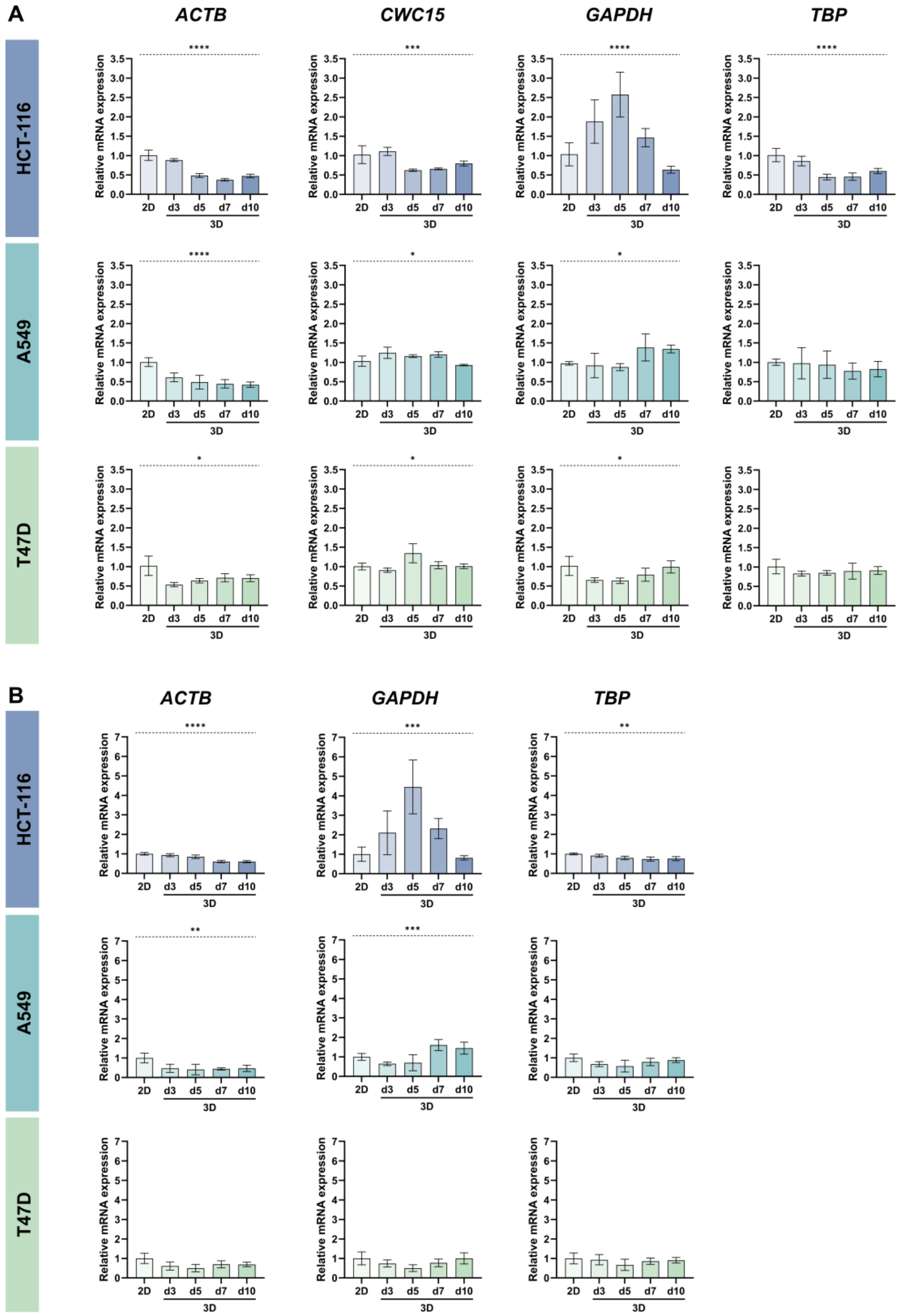

Figure S8: Comparison of housekeeper gene expression analyzed via RT-qPCR.
A. Relative mRNA expression of *ACTB*, *CWC15*, *GAPDH* and *TBP* normalized to 2D for all three cell lines determined by RT-qPCR (*n* = 3 to 4). **B.** Relative mRNA expression of *ACTB*, *GAPDH* and *TBP* normalized to *CWC15* and 2D for all three cell lines determined by RT-qPCR (*n* = 3 to 4). Data shown in A and B are from the same experiment but normalized differently. Data are shown as mean values ± SD. Statistical significance was calculated using one-way ANOVA. The p-value is depicted as stars (* $p < 0.05$, ** $p < 0.01$, *** $p < 0.001$, **** $p < 0.0001$) and describes a significant effect over time.

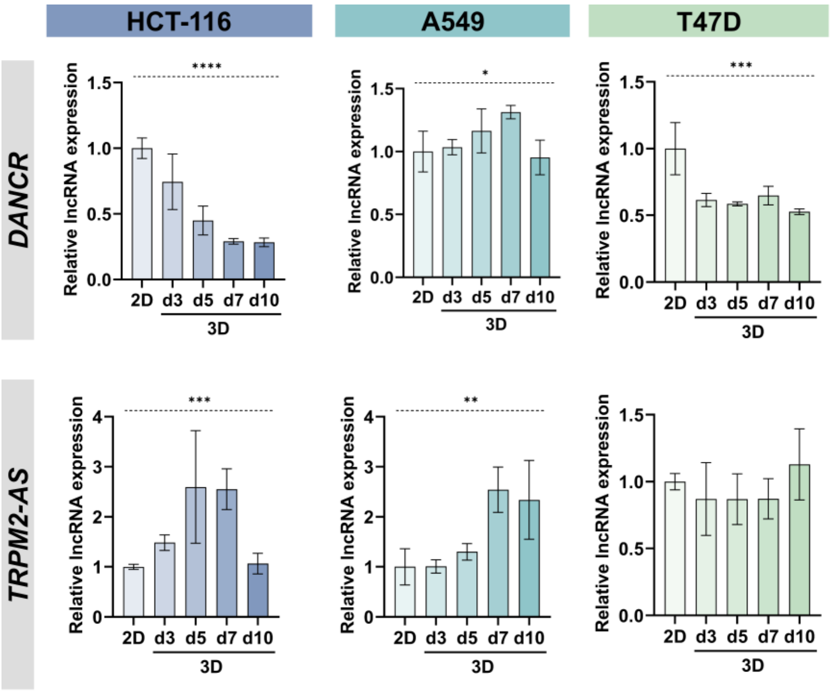

Figure S9: Analysis of key lncRNA gene expression via RT-qPCR.
Relative gene expression of *DANCR* and *TRPM2-AS* normalized to *CWC15* and 2D for all three cell lines determined by RT-qPCR (n = 3). Data are shown as mean values ± SD. Statistical significance was calculated using one-way ANOVA. The p-value is depicted as stars (* $p < 0.05$, ** $p < 0.01$, *** $p < 0.001$, **** $p < 0.0001$) and describes a significant effect over time.

Table S1: Selected cancer-related lncRNAs regulated in HCT-116, A549 or T47D cultivated in 3D Coll I matrices.

Gene symbol	Gene name	ENSEMBL ID
DRAIC	Downregulated RNA in cancer, inhibitor of cell invasion and migration	ENSG00000245750
PRNCR1	Prostate cancer associated non-coding RNA 1	ENSG00000282961
HULC	Hepatocellular carcinoma up-regulated long non-coding RNA	ENSG00000285219
SOX2-OT	SOX2 overlapping transcript	ENSG00000242808
DANCR	Differentiation antagonizing non-protein coding RNA	ENSG00000226950
TRPM2-AS	Transient receptor potential cation channel subfamily M member 2 antisense RNA	ENSG00000230061
DIRC3	Disrupted in renal carcinoma 3	ENSG00000231672
NEAT1	Nuclear paraspeckle assembly transcript 1	ENSG00000245532
MALAT1	Metastasis associated lung adenocarcinoma transcript 1	ENSG00000251562
LINC00665	Long intergenic non-protein coding RNA 665	ENSG00000232677
NKILA	NF-kappaB interacting lncRNA	ENSG00000278709
LINC01410	Long intergenic non-protein coding RNA 1410	ENSG00000238113

Table S2: Cell numbers seeded per well or coverslip for 3D cell cultivation applications.

Cell line	Embedding in 3D Coll I matrix	Invasion assay
**HCT-116**	60’000	8’000
**A549**	30’000	10’000
**T47D**	90’000	13’000
**MDA-MB-231**	-	11’000

Table S3: Primer sequences used for RT-qPCR.

Target	Primer sequence (5'-3')	Primer sequence (5'-3')	Product size (bp)
GAPDH	TGTTCGACAGTCAGCCGCATCT	GTGACCAGGCGCCCAATACGA	117
ACTB	GAGCCTCGCCTTTGCCGATCC	GCACATGCCGGAGCCGTTGT	103
TBP	AACGCCGAATATAATCCCAAGCG	CCTGTGCACACCATTTTCCCAG	101
CWC15	GCAGATGACCCTCTAACAGATGAG	TGGCCTGCTCTTCAGCTCTTTC	124
CDH1	ACTGGGCTGGACCGAGAGAG	GTTGTGCTTAACCCCTCACCTTG	81
DSP	AGAGAGATGCGGCAGATGGGC	TGCGGACTCGAGGGACACTG	112
ITGA2	TCAAGCACAGAGTTGCCCCGA	TAGGCTTCAAGGGCAGGGCT	126
ITGB1	ACCTGCCTTGGTGTCTGTGCTG	TGGACCGGCTGGGGTAATTTGTC	149
CCNA2	GCGCTGGCGGTACTGAAGTC	CCAAGGAGGAACGGTGACATGC	126
CCNB1	TGTGGATGCAGAAGATGGAGCTGAT	TCCAGTGACTTCCCGACCCAGT	133
CCNB2	GCATGCGTGCCATCCTAGTGGA	GCTTCTTCCGGGAAACTGGCTGA	132
CXCR4	CATGGAGGGGATCAGTATATACAC	GCATTTTCTTCACGGAAACAGGG	102
DDIT4	TTGACCGCTCCACGAGCCTG	CATCCAGGTAAGCCGTGTCTTC	96
ANGPTL4	CAGCATCTGCAAAGCCAGTTTG	CATTGTGAGCCGGGTCAACTG	121
